# Saliva as a Potential Source of Biomarkers in Cows with Metritis: A Pilot Study

**DOI:** 10.3390/vetsci11090446

**Published:** 2024-09-21

**Authors:** Pedro J. Vallejo-Mateo, María D. Contreras-Aguilar, Alberto Muñoz-Prieto, María Botia, Asta Tvarijonaviciute, Camila Peres Rubio, Rasa Zelvyte, José J. Cerón, Lorena Franco-Martínez

**Affiliations:** 1 Department of Animal Medicine and Surgery, Veterinary School, Regional Campus of International Excellence Campus Mare Nostrum, University of Murcia, Campus de Espinardo, Espinardo, 30100 Murcia, Spain; pedroja512@hotmail.com; 2 Interdisciplinary Laboratory of Clinical Analysis of the University of Murcia (Interlab-UMU), Department of Animal Medicine & Surgery, Veterinary School, Campus Mare Nostrum, University of Murcia, 30100 Murcia, Spain; alberto.munoz@um.es (A.M.-P.); maria.botiag@um.es (M.B.); asta@um.es (A.T.); camila.peres@um.es (C.P.R.); jjceron@um.es (J.J.C.); lorena.franco2@um.es (L.F.-M.); 3 Department of Anatomy and Physiology, Research Center of Digestive Physiology and Pathology, Veterinary Academy, Lithuanian University of Health Sciences, Tilzes str. 18, LT-47181 Kaunas, Lithuania; rasa.zelvyte@lsmu.lt

**Keywords:** biomarkers, dairy cattle, metritis, oral fluid, saliva, sialochemistry

## Abstract

**Simple Summary:**

Metritis, a condition affecting up to 20% of cows after giving birth, negatively impacts animal welfare and dairy farm profitability by reducing productivity and reproduction. This study compared the biochemical profiles of healthy cows and those with metritis, analyzing 25 salivary and 31 serum analytes alongside various health parameters. Key findings showed that cows with metritis had elevated levels of certain biomarkers related to stress, inflammation, and metabolism in both saliva and serum. Specifically, eight salivary biomarkers including adenosine deaminase (ADA) and haptoglobin (Hp) and eight serum biomarkers including ADA, Hp, and serum amyloid A (SAA) were significantly higher in affected cows. In contrast, six biomarkers including total esterase (TEA) and albumin were lower in the serum. This study highlights the potential of using saliva as a non-invasive source for identifying biomarkers in cows with metritis, offering a new approach to diagnose and manage this condition.

**Abstract:**

Metritis affects 5–20% of cows after parturition, negatively impacting animal welfare and the profitability of dairy farms, increasing culling rates and costs, and decreasing productivity and reproduction rates. This study compared the results of a comprehensive biochemical panel consisting of 25 salivary and 31 serum analytes between healthy cows (*n* = 16) and cows with metritis (*n* = 12). Descriptive parameters such as depression, rectal temperature, body condition score (BCS), heart rate, respiratory rate, mucous color, ruminal motility, vaginal discharge, milk production, and complete hematology analyses were also assessed for comparative purposes. The biochemistry analytes comprised five analytes related to stress, five to inflammation, five to oxidative status, and nineteen to general metabolism. The two-way ANOVA analysis revealed that, in saliva, eight biomarkers (lipase, adenosine deaminase (ADA), haptoglobin (Hp), total proteins, g-glutamyl transferase (gGT), aspartate aminotransferase (AST), alkaline phosphatase (ALP), and creatine kinase (CK)) were significant higher in cows with metritis. In serum, eight biomarkers (ADA, Hp, serum amyloid A (SAA), fibrinogen, ferritin, AOPPs/albumin ratio, non-esterified fatty acids (NEFAs), and bilirubin) were significantly higher in cows with metritis, whereas six (total esterase (TEA), albumin, urea, lactate, phosphorus, and calcium) were lower. Of the total number of 23 biomarkers that were measured in both saliva and serum, significant positive correlations between the two biofluids were found for six of them (Hp, FRAP, CUPRAC, AOPPs, urea, and phosphorus). Urea showed an R = 0.7, and the correlations of the other analytes were weak (R < 0.4). In conclusion, cows with metritis exhibited differences in biomarkers of stress, inflammation, cellular immune system, and general metabolism in both salivary and serum biochemistry profiles. These changes were of different magnitudes in the two biofluids. In addition, with the exception of ADA and Hp, the analytes that showed changes in the saliva and serum profiles of cows affected by metritis were different. Overall, this report opens a new window for the use of saliva as potential source of biomarkers in cows with metritis.

## 1. Introduction

Metritis is a uterine infectious disease that is typically observed in dairy cows during the first 21 days after parturition. The disease is very common, affecting 5–20% of cows [1], and is the second most common cause for antimicrobial treatment in cows, behind mastitis [2]. On dairy farms, metritis leads to reduced milk production and quality [3], increased number of reproductive problems such as delayed uterine involution or reduced pregnancy rate [4,5,6], and increased early cullings [7,8]. Cows with metritis had altered rumination times and physical activity and increased lying times [9]. The reduced welfare of the affected animals is also associated with reduced food intake and contributes to an immunosuppressive state and subsequent associated pathologies.

Although there are some guidelines published in this regard, one of the current challenges with metritis in dairy cows is the misdiagnosis and unnecessary antimicrobial treatment of animals, which promotes antimicrobial resistance [10]. Although external clinical signs such as possible changes in the odor, color, or viscosity of vaginal discharge or the presence of fever are important criteria in the clinical diagnosis of metritis, there is no standardized case definition, and some animals may present subclinical forms, in which these external clinical signs are not evident [10]. Changes in the biochemical profile of cows with metritis have been described previously [4,5].

In recent years, the use of non-invasive samples such as saliva has been receiving increasing attention due to their potential in the diagnosis and follow-up of a variety of physiological and pathological processes [11,12]. Saliva is being used in humans and animals, including cows, for the assessment of stress, inflammation, redox status, and tissue damage or infections, among others [11,13]. There are several studies in which a salivary biochemistry profile comprising analytes that are usually measured in serum and plasma (called “sialochemistry”) has been assessed in cows with different conditions such as mastitis, calving, or lameness, showing differences in comparison with healthy controls [11,12,13]. All of this comes with the main advantages of being easy to collect, inexpensive to sample and process, painless, and producing minimum stress to animals and staff. However, to the best of our knowledge, there are no reports of changes in analytes in the saliva of cows with metritis and about the comparison of possible differences in analytes in saliva and serum in this disease.

The aims of the present study are to evaluate the possible changes observed in a comprehensive panel of salivary and serum biomarkers between healthy cows and cows with metritis. For comparative purposes, complete blood hematology and selected clinical descriptive parameters were included in this report. This study will increase the knowledge about the pathophysiology of this disease and help identify potential biomarkers in saliva and serum for possible future use in the diagnosis and treatment monitoring of metritis.

## 2. Materials and Methods

### 2.1. Animals and Sample Collection

This was a prospective case–control study comprising Holstein Friesian dairy cows from a Spanish commercial dairy herd (38°2′ N, 1°15′ W) and was performed during 12 weeks in the months of March to May. The feeding was based on a total mixed ration and was offered ad libitum, as was water intake. The animals were routinely checked by an experienced veterinarian (Pedro J. Vallejo-Mateo) and milked twice a day. The farm was free of brucellosis, tuberculosis, bovine leukosis virus, and pleuropneumonia. The animals were vaccinated against bovine viral diarrhea, infectious bovine rhinotracheitis, bovine parainfluenza 3, and bovine respiratory syncytial virus.

All the procedures carried out in this study were approved by the Bioethical Committee (CEEA) of the University of Murcia (Spain), under the number 171/2015. Using data from a preliminary trial involving 5 healthy cows and cows with metritis, a sample size calculation was conducted to ensure statistical power for each analyte. The sample size calculation was conducted to ensure a significance level of α = 5% (*p* < 0.05) and a power of 80%. The highest sample size determined was *n* = 8 for each group.

Over the period of this study, a total of *n* = 16 cows were diagnosed with metritis, based on visual inspection and clinical examination (depression, temperature, presence of vaginal discharge, heart rate, respiratory rate) by an experienced veterinarian, as described in Table 1. The criteria for inclusion were as follows: (1) clinical signs compatible with metritis according to the previous literature (enlarged uterus and fetid abnormal uterine discharge, associated with signs of systemic illness such as decreased milk yield, dullness, or other signs of toxemia) within 21 days after parturition [14]; (2) no other complications such as lameness or ketosis, as determined by visual inspection and serum beta-hydroxybutyrate measurements below 0.8 mmol/L, respectively; and (3) not having any clinical evidence of acute or chronic disease other than the current episode of metritis in the last 3 months. For each animal, clinical evaluation and sampling were performed at the moment of diagnosis and prior to any treatment. Of the 16 animals with metritis, 4 had lameness or ketosis at the time of the metritis diagnosis and, therefore, were removed from this study to avoid possible interferences, leaving a total of *n* = 12 animals for the metritis group. A group of *n* = 16 cows from the same herd selected to match in age, lactation number, and days postpartum that did not show any health issues upon visual inspection were also sampled and included in this study as healthy controls (H group). For each cow with metritis, another cow of similar parity was sampled on the same day. Data from the clinical evaluation consisting of depression, body condition score (BCS), temperature, heart rate, respiratory rate, mucosa color, ruminal motility, presence and characteristics of vaginal discharge, and milk yield were collected for all the cows. All the data were referred to the day of each animal’s sampling. The BCS was assessed using a 5-point scale with 0.25-point increments (1 = thin and 5 = fat), as previously described [15]. The depression score was adapted from a previous report [16] and was based on visual inspection of demeanor, ears, mobility, interest in surroundings, feed intake, and enophthalmos using a 5-point scale with 1-point increments, in which 1 was no depression, and 5 indicated severe depression.

The sampling was performed as described in a previous report [12]. In brief, after washing the mouth with water using a feeding tube and waiting for 3 min to avoid possible saliva dilution, a sponge (45 mm × 25 mm × 25 mm; Esponja Marina, La Griega E. Koronis, Madrid, Spain) clipped to a flexible thin metal rod was introduced into the cow’s mouth for salivary collection. Once the sponge was moist, it was placed in a collection tube (Salivette, Sarstedt, Aktiengesellschaft & Co., Nümbrecht, Germany). Then, blood was obtained by venipuncture from the coccygeal vein and stored in tubes with a clotting activator separator gel (Tapval, Aquisel, Barcelona, Spain) to obtain the serum and EDTA for hematology and fibrinogen testing. The saliva and blood were stored refrigerated at 4 °C for less than two hours, until arrival at the laboratory. Finally, the EDTA blood was analyzed for hematology upon arrival, and the saliva and blood were then centrifuged (3000× *g* for 10 min at 4 °C) and stored in Eppendorf tubes for less than five months at −80 °C until biochemical analysis.

### 2.2. Blood and Salivary Analyses

For whole blood, hemograms were performed using an automated analyzer (ADVIA 120 hematology Analyzer; Siemens Healthcare GmbH; Erlangen; Germany). 

In the case of saliva and serum biochemistry, two different types of analysis were made:

(a) Spectrophotometric or turbidimetric assays using an automatic biochemistry analyzer (Olympus AU600, Beckman Coulter, Ennis, Ireland). These were applied for the stress biomarkers: total esterase (TEA), and salivary alpha-amylase, butyryl-cholinesterase (BChE), and lipase (Lip). Also, for the immunity and inflammation biomarkers: adenosine deaminase (ADA) and serum amyloid A (SAA) fibrinogen (measured in EDTA samples), and ferritin. And, in addition, for the oxidative status biomarkers: Trolox equivalent antioxidant capacity (TEAC), ferric reducing antioxidant power assay (FRAP), cupric reducing antioxidant capacity (CUPRAC), uric acid, and advanced oxidation protein products (AOPPs). These methods were also applied for the biomarkers of general metabolism and liver, muscle, and renal damage (enzymes, proteins, and minerals): non-esterified fatty acids (NEFAs), proteins, albumin, bilirubin, creatine kinase (CK), γ-glutamyl transferase (gGT), aspartate aminotransferase (AST), alkaline phosphatase (ALP), creatinine, urea, triglycerides, glucose, lipase, amylase, lactate, lactate dehydrogenase (LDH), phosphorus, calcium, and iron. In all cases, previously validated methods were used.

(b) In-house immunologic methods utilizing AlphaLISA technology (PerkinElmer, Inc., Hopkinton, MA, USA) with a 96-well fluorometry plate reader (PerkinElmer, Inc., Hopkinton, MA, USA) for cortisol and for haptoglobin in saliva (Hp).

The data documenting precision and accuracy for saliva and serum are provided in Supplementary Figure S1, showing that all the assay values were within the acceptance range limits according to the guidance for industry [17].

### 2.3. Statistical Analysis

The data from this study are expressed as median and 25–75th interquartile ranges, unless otherwise stated. For group comparison, the data were first evaluated for the normality of the distribution using the D’Agostino and Pearson omnibus normality tests. As not all the data followed a normal distribution, the data were log transformed or non-parametric tests were performed. Fold changes were expressed as median (metritis) divided by median (healthy controls).

In the ANOVA analysis, the data were log1p transformed and any missing values were imputed with the median for their respective group (healthy/metritis). The effect of the group compared the measurements between healthy cows and cows with metritis. To evaluate whether the differences in analytes could be affected by the BCS, this effect was also evaluated both as an independent factor and through the Group–BCS interaction.

The correlation matrix between all the cytokines was calculated using the non-parametric Spearman correlation test.

In all cases, the differences were considered significant if the *p*-value was below 0.05. Statistics were performed using RStudio (RStudio Team, Version: 2024.04.2+764).

## 3. Results

### 3.1. Descriptive Parameters and Hematology

In the descriptive parameters (Table 1 and Supplemental Figure S1), higher depression scores (1.5-fold) and heart (1.2-fold) and respiratory (1.8-fold) rates were observed in the metritis group, whereas milk yield (0.7-fold) and ruminal motility (1-fold) were lower in the metritis group.

The ANOVA results for the hematological parameters (Table 1 and Supplemental Figure S2) revealed that the levels of eosinophils were 0.7-fold lower in cows with metritis. The eosinophils were observed to be affected significantly due to the Group–BCS interaction.

When the possible effects of the BCS and the Group–BCS interaction were assessed for salivary and serum biomarkers (Supplemental Figures S3 and S4), in saliva, the uric acid and proteins were significantly different according to the BCS, and no biomarkers differed significantly for the Group–BCS interaction. For serum, only SAA was affected significantly by the BCS, and no significant changes were observed in serum for the Group–BCS interaction.

### 3.2. Salivary Biomarkers

The data from the salivary and serum analytes, including ANOVA effects for the group comparison (healthy versus metritis), and correlations between saliva and serum are shown in Table 2 and Table 3 and Supplementary Figure S3.

The results for sialochemistry and ANOVA in saliva are shown in Supplementary Figure S3. On the salivary biomarkers, the two-way ANOVA analysis revealed that “Group” (healthy vs. metritis) significantly affected eight biomarkers. The levels of lipase (the healthy group had a median of 0 and the metritis group showed 23.26 (UI/L), ADA (1.76-fold higher), Hp (6.27-fold higher), proteins (1.26-fold), gGT (2.46-fold), AST (3.68-fold), ALP (5.14-fold), and CK (2.01-fold)) were higher in cows with metritis.

### 3.3. Serum Biochemistry Biomarkers

The two-way ANOVA analysis on serum biochemistry biomarkers (Table 2 and Table 3, and Supplementary Figure S4) revealed that fourteen analytes were significantly different between healthy cows and cows with metritis. The levels of ADA (1.4-fold), Hp (the healthy group had a median of 0 and the metritis group showed 0.6 g/L), SAA (18.3-fold), fibrinogen (3.6-fold), ferritin (1.7-fold), AOPPs/albumin ratio (1.36-fold), NEFAs (2.2-fold), and bilirubin (1.6-fold) were higher in metritis. In contrast, TEA (0.7-fold), albumin (0.8-fold), urea (0.6-fold), lactate (0.9-fold), phosphorus (0.9-fold), and calcium (0.9-fold) were lower in the metritis group.

### 3.4. Correlation between Serum and Saliva Results

When the correlations among the measurement of the same biomarkers in saliva and serum were evaluated (Table 2 and Table 3), positive correlations were found for Hp, FRAP, CUPRAC, AOPPs (with serum AOPP/albumin ratio), urea, and phosphorus. Of those, only urea showed a strong correlation coefficient with R > 0.7.

## 4. Discussion

In this report, it is described that cows with metritis show differences in saliva and serum analytes compared with healthy cows. The panel of analytes used in this study was chosen to cover different physiological pathways such as stress, immunity and inflammation, and redox status [11,12,13] to obtain a general overview of the differences that can be produced in these systems in cows with metritis at the moment of diagnosis. For this, a total of 25 salivary and 31 serum biomarkers were employed. Since not all the methods were analytically validated for use in saliva or serum, some of the analytes were measured in serum but not in saliva, and vice versa, as depicted in Table 2 and Table 3. To facilitate their inclusion in routine clinical analysis, all analytes included in this study, except for cortisol in serum and saliva and Hp in saliva can be automatically measured.

In-farm descriptives are of high importance for the clinical diagnosis of metritis since they are the first clue to the veterinarian to suspect metritis at puerperium. In this study, the cows with metritis had a higher depression score, heart rate, respiratory rate, and ruminal motility than the controls (1.5-, 1.2-, 1.8-, and 1-fold higher, respectively), and a lower milk yield (0.7-fold). Interestingly, no significant changes were found for rectal temperature, which is a common symptom of metritis [10], although different studies report about 80–50% of animals not having fever (>39.5 °C) in this disease [18,19]. The results for milk agreed with the previous literature that reports lower milk yield in cows with metritis when compared with healthy ones [4,14,18,20]. Overall, the differences observed in the farm descriptives were compatible with the presence of metritis.

In our study, the hematological analyses did not show significant differences between healthy cows and cows with metritis, except for a 0.7-fold lower eosinophil count in metritis. In addition, a decrease in the lymphocytes (0.9-fold) was observed (although it was not statistically significant), which has been described in cows with metritis [18,19]. These findings could be compatible with a stress leukogram, which typically includes eosinopenia and lymphopenia.

### 4.1. Biomarkers of Stress, Immune System, and Inflammation

The biomarkers of stress used in our study were serum and salivary cortisol and TEA and salivary BChE, alpha-amylase, and lipase. Serum butyrylcholinesterase was not measured in the ruminants, since its activity is very low [21]. In our report, significant increases in cows with metritis were found for lipase in saliva. This is in line with increases in lipase in saliva that have been previously reported in cows with other inflammatory conditions such as lameness [13] and with the correlation that lipase showed with inflammation during peripartum [12]. No significant differences were found for cortisol in our study, which is in line with previous reports [19,22]. In the case of salivary alpha-amylase, a biomarker of sympathetic activation, although it was not statistically significant, two-fold higher mean values were observed in diseased cows. Therefore, our findings suggest that cows with metritis could have higher stress than healthy controls, probably because of the inflammatory status and the discomfort experienced during the disease.

A panel integrated by Hp, SAA, fibrinogen, and ferritin as inflammatory biomarkers was employed. These are acute-phase proteins (APPs), which are biomarkers that are very sensitive to inflammation, varying their concentrations rapidly after any tissular damage. In cows, Hp and SAA are considered major APPs, whereas fibrinogen and ferritin can be considered as moderate or minor APPs [23]. In this study, all the APPs measured exhibited statistically higher values in cows with metritis in comparison with healthy ones. The Hp in saliva was 6.3-fold higher, and the serum revealed increases for SAA (18.3-fold), fibrinogen (3.6-fold), and ferritin (1.7-fold). In addition, for serum Hp, all healthy animals and 25% of cows with metritis were below the assay’s lower limit of quantification (LLOQ), which agrees with a previous study using plasma in which only the most affected animals exhibited a strong haptoglobin response [24]. This was not observed in the case of saliva, in which all cows with metritis and 43% of healthy animals exhibited Hp values above the LLOQ. Thus, it could be postulated that, in the case of Hp, saliva could be more sensitive than serum for the detection of inflammation associated with metritis.

Adenosine deaminase, a biomarker of activation of the immune system and cell mediated activity [25], was also increased in both saliva (1.8-fold) and serum (1.4-fold) in cows with metritis compared with healthy ones. ADA concentrations were higher in serum than in saliva as reported previously [26] but increases of higher magnitude in ADA in saliva than in serum were observed in our study as reported in other inflammatory conditions [26]. This biomarker has also been proposed as a marker of proinflammatory status in domestic species, being found elevated in the serum of cows after calving [27].

### 4.2. Biomarkers of Redox Status Stress; General Metabolism; And Liver, Muscle, and Renal Damage

In this study, oxidative stress was assessed through TEAC, FRAP, CUPRAC, and uric acid in serum and saliva and through AOPPs (AOPPs in saliva and AOPP/albumin ratio in serum, as reported elsewhere [28]), and no significant changes in these analytes between the cows with metritis and healthy cows were found. Other studies reported altered reactive oxygen metabolites (d-ROMs), antioxidants (OXYs), and oxidative status index (OSI) levels in plasma in cows with metritis [20] and higher malondialdehyde and lower vitamins A and E in the serum of cows with metritis [18]. In other infectious processes such as mastitis, increases in salivary levels for uric acid and in serum levels for uric acid and CUPRAC were also reported [11]. Further studies should be performed to determine these discrepancies, although these divergences could be due to factors such as possible differences in the severity of the disease or sample size.

Finally, in the group of biomarkers of general metabolism and liver, muscle, and renal damage, several differences were observed in saliva and serum between healthy and diseased animals. Five biomarkers in saliva (proteins (1.3-fold), gGt (2.5-fold), AST (3.7-fold), ALP (5.1-fold), and CK (2.01-fold)) were increased in cows with metritis. In the serum, NEFAs (2.2-fold) and bilirubin (1.6-fold) were higher in metritis. However, serum albumin (0.8-fold), urea (0.6-fold), lactate (0.9-fold), phosphorus (0.9-fold), and calcium (0.9-fold) were decreased in comparison with controls. These results agree with the previous literature in which lower serum levels for albumin, urea and Ca were reported in cows with metritis, as well as higher NEFAs [18,29] and bilirubin [5,18,19,30,31]. The insufficient energy intake in the periparturient period promotes lipomobilization and increased NEFAs [18,32] as well as liver damage [18,32,33], which can be reflected by the increase in bilirubin, gGT, AST, and ALP observed in our study for saliva and in the case of serum bilirubin. In addition, the hypocalcemia observed in the serum has been reported to increase the incidence of infectious diseases such as metritis [18,34,35].

To assess the potential impact of the body condition score (BCS) on the panel of salivary and serum biomarkers under investigation, this variable was included in the ANOVA analysis. Additionally, the interaction term Group–BCS was included to examine whether the BCS could have differential effects on the biomarkers depending on the health status of the animals. For serum biomarkers, only Hp was found to be significantly affected by the BCS, exhibiting lower levels in cows with a higher BCS; this could be interpreted by the idea that a low Hp will indicate a low immune system activation and, therefore, more protein available for increasing the BCS. In saliva, uric acid and proteins were also observed to be increased in cows with a higher BCS, being biomarkers that are known to be increased by food intake.

### 4.3. Correlation between Saliva and Serum Biomarkers

This study also evaluated the correlations between all the biomarkers measured in both serum and saliva to determine the relationship between these two biofluids. It was observed that only urea showed a strong positive correlation between saliva and serum, which has also been previously reported for dogs and humans, suggesting that urea enters into the saliva from the blood by passive diffusion through the acini of the salivary glands [36,37]. In addition, positive significant correlations were observed for Hp, FRAP, CUPRAC, AOPPs (AOPPs measured in saliva with serum AOPP/albumin ratio), and phosphorus, although their strength was weak (r from 0.28 to 0.397).

When comparing the results between the salivary and serum biochemical profiles in the two groups of animals, several hypotheses emerge. Saliva demonstrated greater sensitivity in detecting alterations in hepatic biomarkers (proteins, gGT, AST, and ALP), ADA and Hp, whereas variations in urea, lactate, and ions (phosphorus and calcium) were significant only in the serum, indicating that the observations in the serum did not correspond to the observations in the saliva, and vice versa. This is in line with previous reports, in which differences between serum and salivary analysis were observed in cows with different conditions [11]. Therefore, based on these results, it could be indicated that saliva can provide additional information to that provided by the serum, and, therefore, both samples can be used as complementary tools.

### 4.4. Study Limitations

The present study has some limitations. Although the sample size demonstrated enough statistical power in the preliminary and post hoc analyses, it was relatively low, and, thus, it could be considered as a pilot study that needs further verification in larger cohorts. Further validation of the differences observed between healthy cows and cows with metritis is recommended with a larger number of cows and ROC curves. Second, only dairy cows from a particular breed and farm were enrolled, and, therefore, the possible influences of breed, energy status, or handling patterns (i.e., housed, milking, or nutrition handling) were not evaluated. Further studies should be performed to determine whether the biomarkers analyzed here could also be of interest for prevention or as a predictive biomarker of metritis.

## 5. Conclusions

Overall, it can be concluded that metritis in cows is associated with alterations in saliva and serum biomarkers. Saliva analyses revealed increases in 8 biomarkers, while the serum showed significant changes in 14 biomarkers in cows with metritis. These differences point out alterations in stress, inflammation, immune system, and general metabolism. In addition, the saliva showed changes that were different from the ones in the serum, and this could potentially have applications in the diagnosis and monitoring of the disease.

## Figures and Tables

**Table 1:** Median and interquartile (25–75th percentile) data of descriptive and hematology parameters of healthy cows (control group, *n* = 16) and cows with metritis (metritis group; *n* = 12). Bold highlights *p* < 0.05.

Variable	Healthy Group (*n* = 16)	Metritis Group (*n* = 12)	*p*
Age, years	4 (4–4.75)	4.5 (4–5)	0.420
Days in milk	12 (8–18)	16 (11.25–19)	0.113
Lactation, number	3 (3–4)	3 (2–6)	0.756
Milk yield (Kg/day)	27.5 (19.25–36.5)	19.5 (5.75–30.5)	**0.024**
Body condition score (1–5)	3 (2.19–3)	3 (2.75–3)	0.079
Depression score (1–5)	1 (1–1)	2 (1–3)	**0.001**
Temperature (°C)	38.55 (38.4–38.7)	38.8 (38.4–39.2)	0.224
Heart rate (bpm)	55 (48–62)	64 (60–78)	**0.003**
Respiratory rate (rpm)	24 (18.25–31)	36 (31–56)	**0.006**
Ruminal motility (1–3)	2 (2–3)	2 (1–2)	**0.028**
Hematocrit (%)	26.2 (25.05–27.55)	24.9 (20.93–27.88)	0.170
White blood cells (WBCs, ×10^3^ µL)	7.37 (5.32–8.13)	5.28 (4.69–10.22)	0.478
Neutrophils (×10^3^ µL)	2.91 (2.41-4.59)	1.92 (1.08–5.4)	0.448
Lymphocytes (×10^3^ µL)	2.86 (2.18–3.7)	2.5 (1.97–3.35)	0.464
Monocytes (×10^3^ µL)	0.37 (0.26–0.56)	0.47 (0.25–0.62)	0.558
Eosinophils (×10^3^ µL)	0.34 (0.18–0.53)	0.18 (0.11–0.27)	**0.025**
Platelets (×10^3^ µL)	444.5 (346.5–513.25)	457 (267–596.5)	0.621

Body condition score (BCS, 1 = thin and 5 = fat [15]), depression score (1 = no depression and 5 = severe depression), heart rate (beat per minute (bpm)), respiratory rate (respiration per minute (rpm)), ruminal motility (1 = low motility, 3 = high motility).

**Table 2:** Results as median (25–75th interquartile range) data in saliva and serum biomarkers of stress, immunity, and inflammation for healthy controls and cows with metritis, together with results from the two-way analysis of variance (two-way ANOVA, in which “Group” is the factors considered in the analysis) and correlation coefficients (R, P) between the two biofluids, when applicable. Bold highlights *p* < 0.05.

	Saliva			Serum			Correlation Saliva–Serum	
	Healthy	Metritis	Group (P)	Healthy	Metritis	Group (P)	R	P
Cortisol (ng/mL)	40.5 (34.7–57.1)	45.6 (27.8–88.6)	0.790	300 (267–326.9)	300 (291–339)	0.518	0.159	0.516
TEA (UI/L)	168.65 (146.92–213.63)	205.54 (163.62–219.7)	0.976	6.23 (5.32–6.89)	4.34 (3.34–4.68)	**0.005**	0.244	0.095
BChE (µmol/mL/min)	11.23 (8.16–14.97)	13.72 (9.43–16.35)	0.912	-	-	-	-	-
Amylase (UI/L)	2.9 (1.6–3.95)	5.7 (3.6–15.1)	0.126	126.85 (102.48–140.88)	84.45 (65.7–115.45)	0.079	0.025	0.85
Lipase (UI/L)	0 (0–8.48)	23.26 (8.89–50.2)	**0.001**	79.4 (3.83–169.65)	10.95 (1.74–55.35)	0.349	−0.165	0.212
ADA (UI/L)	4.35 (3.72–6.4)	7.66 (4.35–10.06)	**0.032**	6.02 (4.6–7.23)	8.24 (4.89–10.61)	**0.039**	0.199	0.133
Hp (mg/L saliva, g/L serum)	15.35 (7.98–69.96)	96.22 (51.79–345.16)	**0.018**	0 (0–0)	(0.6 (0-1.4))	**0.001**	0.28	**0.038**
SAA (µg/mL)	-	*-*	-	1.85 (1.63–2.9)	33.8 (18.9–71.5)	**0.001**	-	*-*
Fibrinogen (mg/dL)	-	*-*	-	41.69 (23.06–82.11)	149.5 (84.75–644.56)	**0.001**	-	*-*
Ferritin (µg/L)	-	*-*	-	55 (42–62)	95 (37.25–315.25)	**0.036**	-	*-*

Total esterase (TEA), butyryl-cholinesterase (BChE), adenosine deaminase (ADA), haptoglobin (Hp), serum amyloid A (SAA).

**Table 3:** Results as median (25–75th interquartile range) data in saliva and serum biomarkers of oxidative status; general metabolism; and liver, muscle, and renal damage (enzymes, proteins, and minerals) for healthy controls and cows with metritis, together with results from the two-way analysis of variance (two-way ANOVA, in which “Group” is the factors considered in the analysis) and correlation coefficients (R, P) between the two biofluids, when applicable. Bold highlights *p* < 0.05.

	Saliva			Serum			Correlation Saliva–Serum	
	Healthy	Metritis	Group (P)	Healthy	Metritis	Group (P)	R	P
TEAC (mmol/L)	0.56 (0.29–1.06)	0.52 (0.35–0.99)	0.966	0.74 (0.72–0.77)	0.75 (0.6–0.86)	0.775	0.232	0.088
FRAP (mmol/L)	0.72 (0.4–1.53)	0.59 (0.41–1.59)	0.866	0.43 (0.37–0.48)	0.4 (0.32–0.45)	0.710	0.397	**0.003**
CUPRAC (mmol/L)	0.45 (0.25–0.87)	0.4 (0.25–1.04)	0.904	0.53 (0.49–0.56)	0.5 (0.44–0.53)	0.396	0.283	**0.036**
Uric acid (mg/dL)	0.25 (0.25–0.25)	0.25 (0.25–0.29)	0.110	1.12 (0.93–1.21)	1.01 (0.81–1.22)	0.517	−0.112	0.403
AOPPs * (µmol/L)	376.85 (185.63–652)	441 (360.03–846.23)	0.191	0.11 (0.09–0.14)	0.15 (0.09–0.2)	**0.090**	0.295	**0.029**
NEFAs (mmol/L)	-	-	-	0.13 (0.08–0.21)	0.28 (0.18–0.5)	**0.001**	-	-
Proteins (mg/dL saliva, g/dL serum)	46.38 (27.83–62.71)	58.54 (50.52–122.75)	**0.004**	7.44 (6.68–7.87)	6.68 (6.04–7.96)	0.133	0.008	0.954
Albumin (g/dL)	-	-	-	3.44 (3.28–3.67)	2.87 (2.37–3.12)	**0.001**	-	-
Bilirubin (mg/dL)		-	-	0.16 (0.13–0.19)	0.26 (0.14–0.35)	**0.001**	-	-
gGT (UI/L)	10.7 (7.33–13.43)	26.3 (12.8–51)	**0.001**	25.55 (19.4–29.98)	20.25 (16.23–35.03)	0.551	−0.016	0.905
AST (UI/L)	8.5 (5.85–22.68)	31.3 (17.3–153.3)	**0.016**	87.3 (74.63–99.13)	111.6 (81.25–128.18)	0.068	0.203	0.126
ALP (UI/L)	3.5 (1.5–7.83)	18 (6.9–21.7)	**0.002**	37.6 (28.7–40.8)	36.7 (28.63–55.95)	0.285	−0.083	0.533
CK (UI/L)	1.7 (1.07–3.13)	3.33 (1.71–3.13)	**0.021**	135.65 (107.1–164.78)	136.9 (97.93–296.88)	0.692	0.23	**0.088**
Creatinine (mg/dL)	0.52 (0.39–0.83)	0.57 (0.34–0.91)	0.931	0.86 (0.72–0.89)	0.86 (0.69–0.95)	0.616	0.084	0.526
Urea (mg/dL)	21.9 (19.93–25.53)	22.5 (17.6–32.7)	0.519	24 (17.23–28.53)	15.3 (12.95–23.78)	**0.009**	0.705	**0.001**
Triglycerides (UI/L)	56.21 (27.16–169.86)	34.52 (18.42–64.27)	0.415	11.36 (9.34–12.5)	9.44 (7.92–11.77)	0.304	−0.047	0.724
Glucose (mg/dL)	26.55 (11.71–97.9)	20.86 (5.4–99.3)	0.628	57.75 (51.3–62.63)	55.5 (51.95–60)	0.924	−0.191	0.152
Lactate (mmol/L)	3.66 (2.59–9.72)	8.15 (2.16–11.03)	0.997	1.15 (0.92–1.46)	0.98 (0.79–1.21)	**0.015**	0.115	0.389
LDH (UI/L)	38.65 (21.35–82.4)	42.8 (25.9–78.5)	0.349	1975 (1813.8–2053.83)	2243.1 (2076.73–2445.78)	0.057	−0.036	0.789
Phosphorous (mg/dL)	25.13 (21.46–28.28)	19.43 (15.43–25.4)	0.069	6.24 (5.56–6.61)	5.5 (4.67–5.88)	**0.048**	0.29	**0.027**
Calcium (mg/dL)	2.62 (1.78–5.38)	2.52 (1.41–4.99)	0.620	12.49 (11.4–12.87)	10.88 (9.03–11.2)	**0.003**	0.081	0.546
Iron (µg/L)	-	-	-	101.5 (88.68–113.6)	107 (83.33–120.18)	0.172	-	-

Trolox equivalent antioxidant capacity (TEAC), ferric reducing antioxidant power assay (FRAP), cupric reducing antioxidant capacity (CUPRAC), advanced oxidation protein products (AOPPs), non-esterified fatty acids (NEFAs), γ-glutamyl transferase (gGT), aspartate aminotransferase (AST), alkaline phosphatase (ALP), creatine kinase (CK), lactate dehydrogenase (LDH). * AOPPs in serum were expressed as the AOPP/Albumin ratio.

## Data Availability

The datasets used in this study are available from the corresponding author upon reasonable request.

## Supplementary Materials

The following supporting information can be downloaded at https://www.mdpi.com/article/10.3390/vetsci11090446/s1: Figure S1. Results of descriptive parameters, consisting of milk yield (kg/day), body condition score (BCS, 1-5), Depression level (from 1 in animals without depression to 3 for animals with severe depression), temperature (in ºC), heart rate (beat per minute (BPM)), respiratory rate (respiration per minute (RPM)), and ruminal motility (movements/minute). Healthy controls are represented in green, while cows with metritis are represented in orange. The plots show the median and 25-75th percentile. Asterisk indicates the statistically significant differences between results (two-way analysis of variance (ANOVA), where "Group", "BSC" and the interaction “Group:BCS” are the factors considered in the analysis) (*: *p* < 0.05; **: *p* < 0.01). Figure S2. Hematology biomarkers in healthy controls (green) and cows with metritis (orange). The plots show the median and 25-75th percentile. Asterisk indicates the statistically significant differences between results (two-way analysis of variance (ANOVA), where "Group", "BSC", and the interaction “Group:BCS” are the factors considered in the analysis) (*: *p* < 0.05). Figure S3. Results in saliva biomarkers of stress (cortisol, alpha-amylase, total esterase (TEA), lipase), immunity and inflammation (adenosine deaminase (ADA), haptoglobin (Hp)), oxidative status (Trolox equivalent antioxidant capacity (TEAC), ferric reducing ability of saliva (FRAS), cupric reducing antioxidant capacity (CUPRAC), uric acid, advanced oxidation protein products (AOPPs)), enzymes (aspartate aminotransferase (AST), alkaline phosphatase (ALP), γ-glutamyl transferase (gGT), lactate dehydrogenase (LDH), creatine kinase (CK)), and proteins and minerals (creatinine, urea, triglycerides, glucose, lactate, proteins, phosphorus, calcium). Healthy controls are represented in green, while cows with metritis are represented in orange. The plots show the median and 25-75th percentile. Asterisk indicates the statistically significant differences between results (two-way analysis of variance (ANOVA), where "Group", "BSC" and the interaction “Group:BCS” are the factors considered in the analysis) (*: *p* < 0.05; **: *p* < 0.01). Figure S4. Results in serum biochemistry biomarkers of stress (cortisol, total esterase (TEA), amylase, lipase), immunity and inflammation (adenosine deaminase (ADA), haptoglobin (Hp), Serum Amyloid A (SAA), Fibrinogen, Ferritin), oxidative status (Trolox equivalent antioxidant capacity (TEAC), ferric reducing antioxidant power assay (FRAP), cupric reducing antioxidant capacity (CUPRAC), uric acid, advanced oxidation protein products/albumin ratio (AOPPs/Albumin)), general metabolisms and enzymes (NEFAs, albumin, bilirubin, aspartate aminotransferase (AST), alkaline phosphatase (ALP), γ-glutamyl transferase (gGT), lactate dehydrogenase (LDH), creatine kinase (CK)), and proteins and minerals (creatinine, urea, triglycerides, glucose, lactate, proteins, phosphorus, calcium and iron). Health controls are represented in green, while cows with metritis are represented in orange. Asterisk indicates the statistically significant differences between results (two-way analysis of variance (ANOVA), where "Group", "BSC" and the interaction “Group:BCS” are the factors considered in the analysis) (*: *p* < 0.05; **: *p* < 0.01).
