# Supplementary material for: Saliva as a Potential Source of Biomarkers in Cows with Metritis: A Pilot Study
Source: Vet Sci. 2024 Sep 21;11(9):446. doi: 10.3390/vetsci11090446 (PMC11435728; doi:10.3390/vetsci11090446)
Supplement: Supplementary file 1 [file vetsci-11-00446-s001.zip › vetsci-3180945-supplementary.pdf]

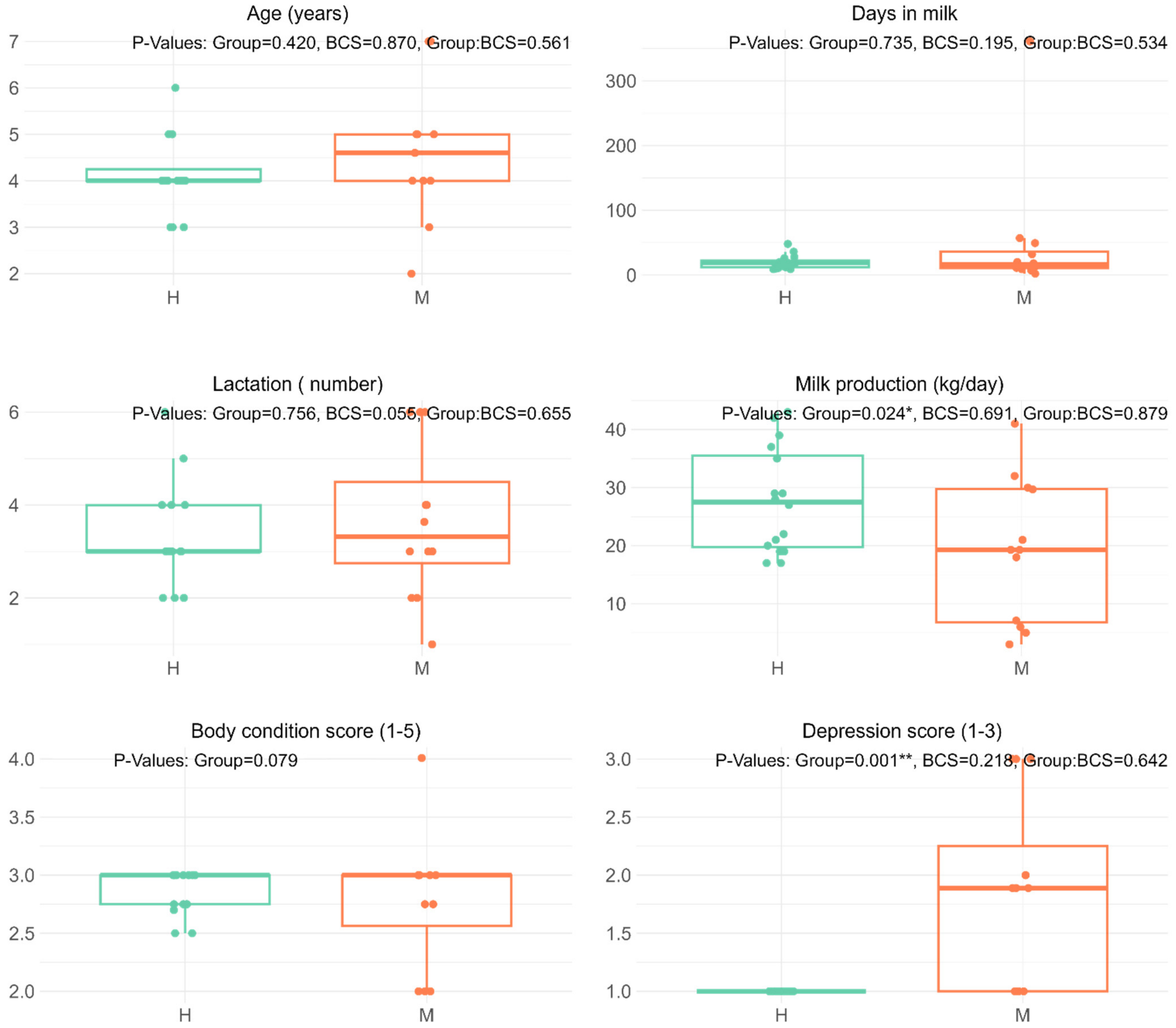
Age (years)
P-Values: Group=0.420, BCS=0.870, Group:BCS=0.561
7
6
5
4
3
2
H
M
Days in milk
P-Values: Group=0.735, BCS=0.195, Group:BCS=0.534
300
200
100
0
H
M
Lactation ( number)
P-Values: Group=0.756, BCS=0.055, Group:BCS=0.655
6
4
2
H
M
Milk production (kg/day)
P-Values: Group=0.024*, BCS=0.691, Group:BCS=0.879
40
30
20
10
H
M
Body condition score (1-5)
P-Values: Group=0.079
4.0
3.5
3.0
2.5
2.0
H
M
Depression score (1-3)
P-Values: Group=0.001**, BCS=0.218, Group:BCS=0.642
3.0
2.5
2.0
1.5
1.0
H
M

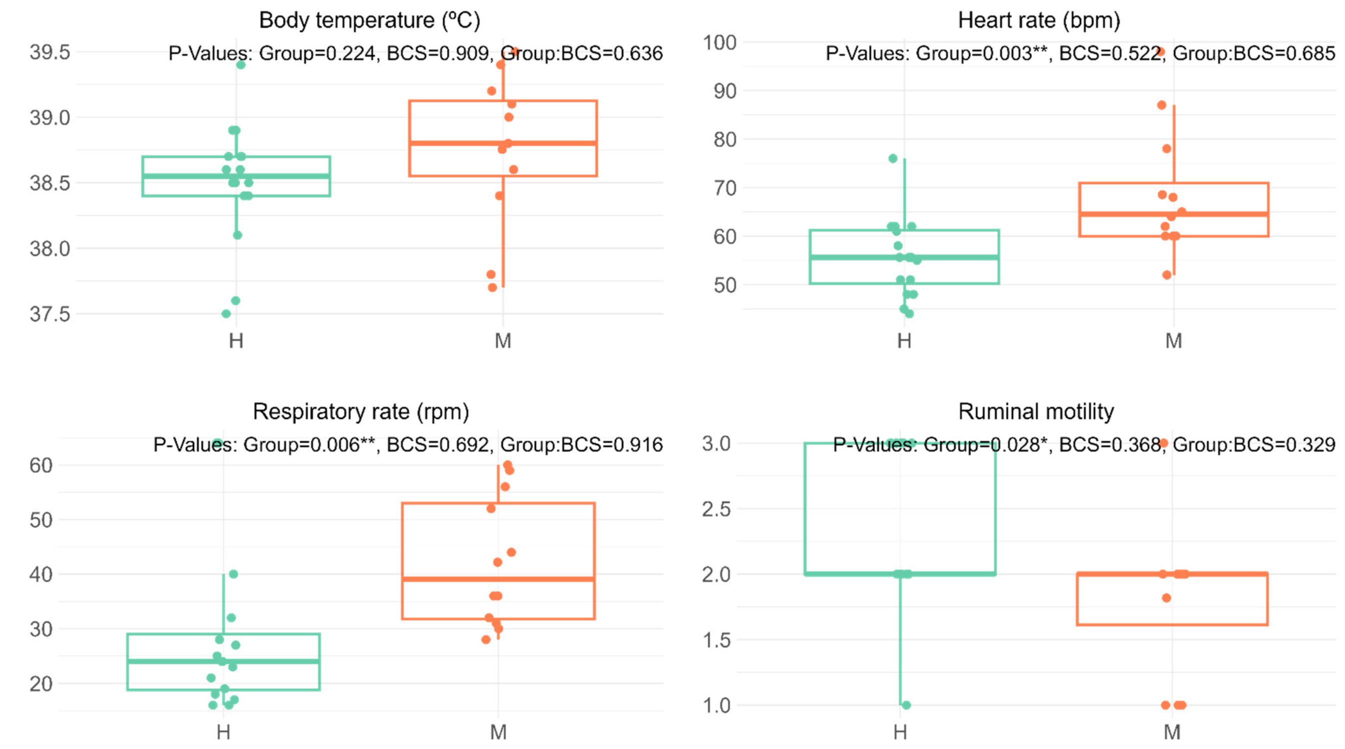


Figure S1. Results of descriptive parameters, consisting of milk yield (kg/day), body condition score (BCS, 1-5), Depression level (from 1 in animals without depression to 3 for animals with severe depression), temperature (in ºC), heart rate (beat per minute (BPM)), respiratory rate (respiration per minute (RPM)), and ruminal motility (movements/minute). Healthy controls are represented in green, while cows with metritis are represented in orange. The plots show the median and 25-75th percentile. Asterisk indicates the statistically significant differences between results (two-way analysis of variance (ANOVA), where "Group", "BSC" and the interaction “Group:BCS” are the factors considered in the analysis) (*: P < 0.05; **: P < 0.01).

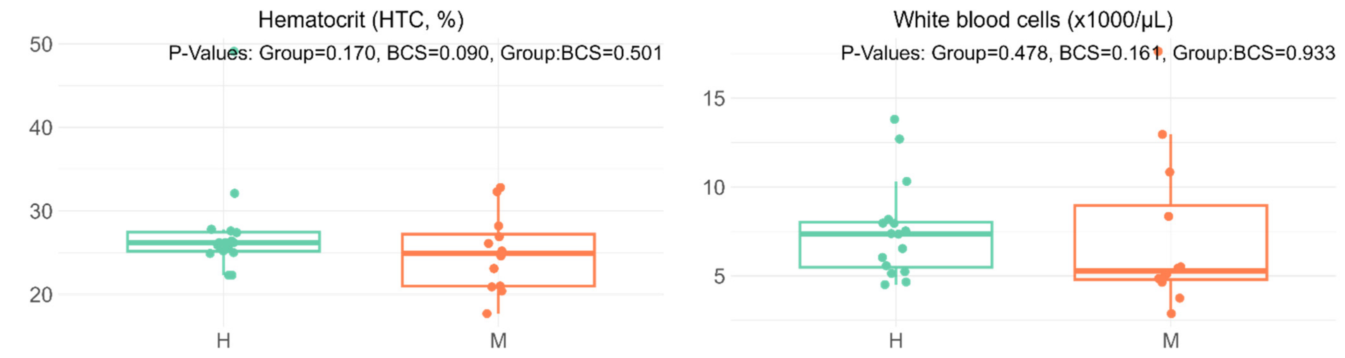

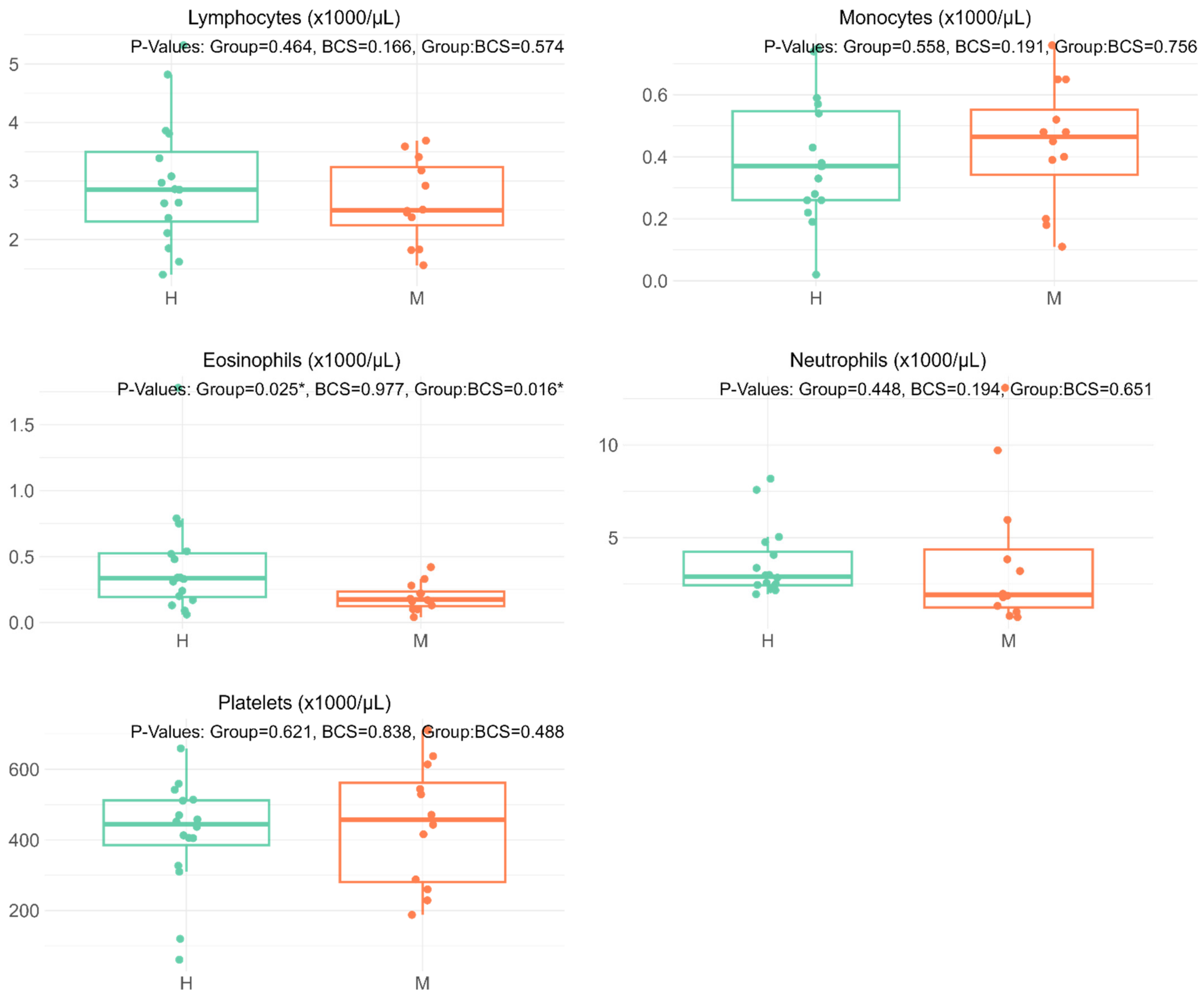


Figure S2. Hematology biomarkers in healthy controls (green) and cows with metritis (orange). The plots show the median and 25-75th percentile. Asterisk indicates the statistically significant differences between results (two-way analysis of variance (ANOVA), where "Group", "BSC", and the interaction “Group:BCS” are the factors considered in the analysis) (*: $P < 0.05$).

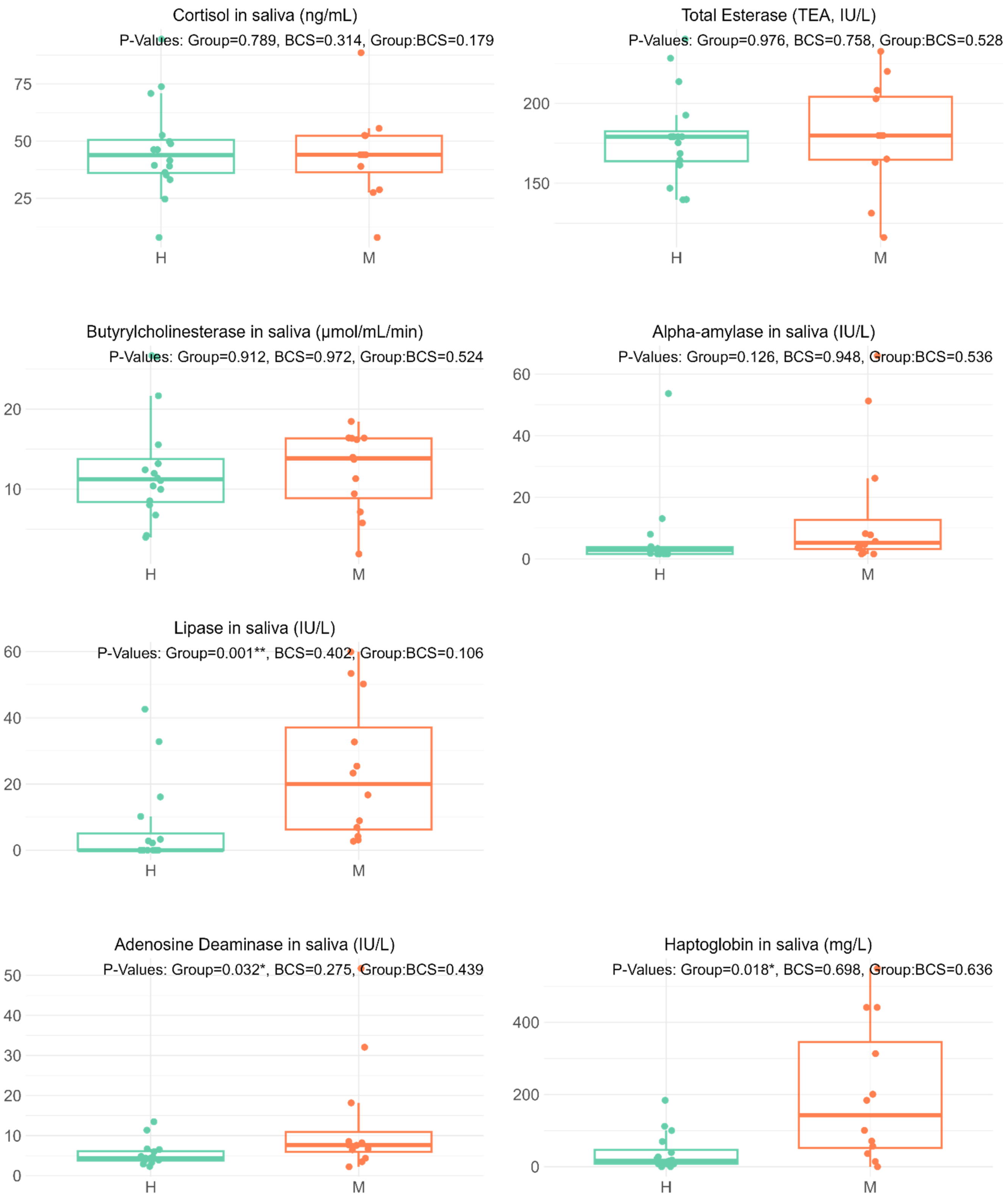

Cortisol in saliva (ng/mL)
P-Values: Group=0.789, BCS=0.314, Group:BCS=0.179
75
50
25
H
M
Total Esterase (TEA, IU/L)
P-Values: Group=0.976, BCS=0.758, Group:BCS=0.528
200
150
H
M
Butyrylcholinesterase in saliva (µmol/mL/min)
P-Values: Group=0.912, BCS=0.972, Group:BCS=0.524
20
10
H
M
Alpha-amylase in saliva (IU/L)
P-Values: Group=0.126, BCS=0.948, Group:BCS=0.536
60
40
20
0
H
M
Lipase in saliva (IU/L)
P-Values: Group=0.001**, BCS=0.402, Group:BCS=0.106
60
40
20
0
H
M
Adenosine Deaminase in saliva (IU/L)
P-Values: Group=0.032*, BCS=0.275, Group:BCS=0.439
50
40
30
20
10
0
H
M
Haptoglobin in saliva (mg/L)
P-Values: Group=0.018*, BCS=0.698, Group:BCS=0.636
400
200
0
H
M

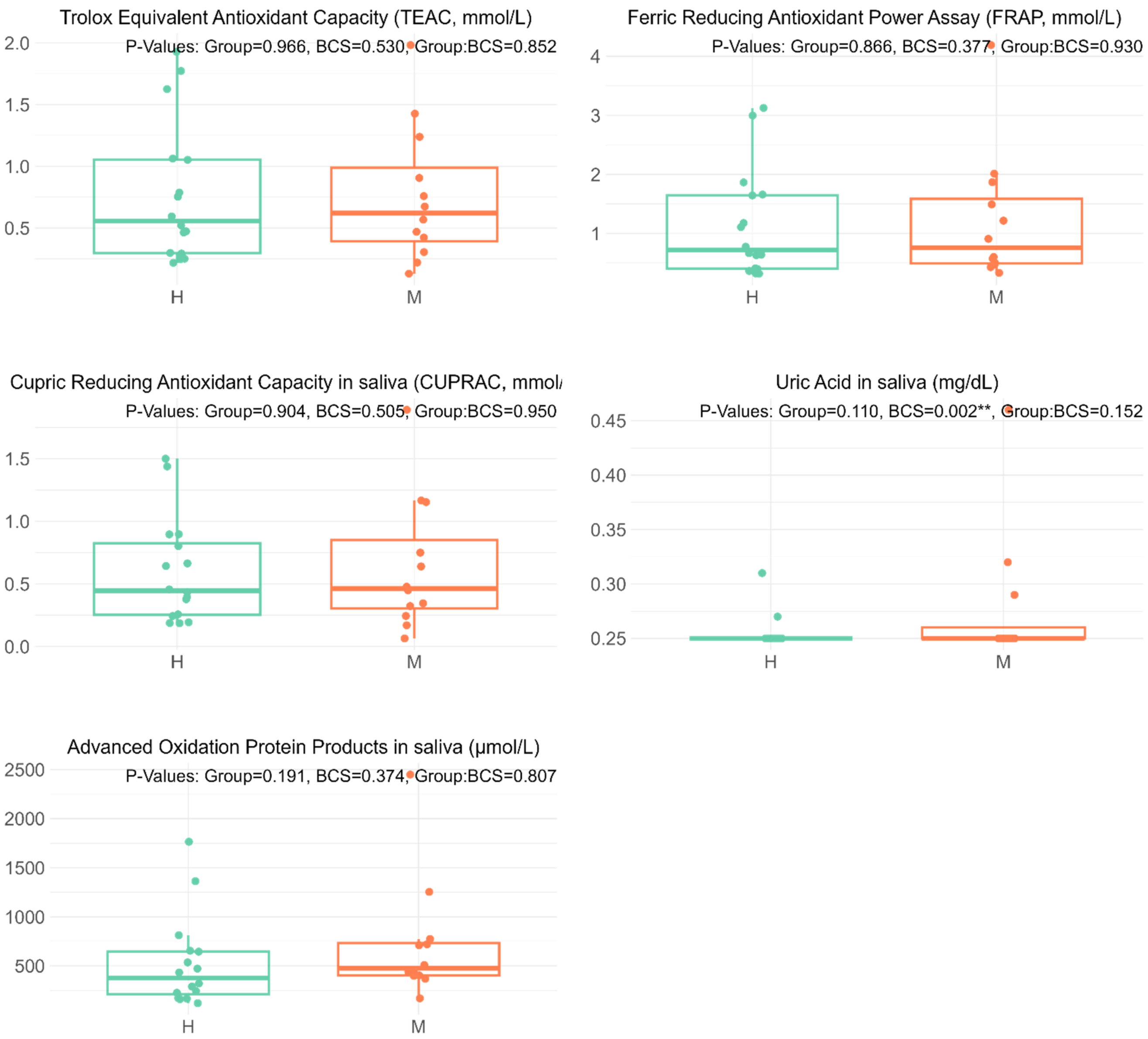
Trolox Equivalent Antioxidant Capacity (TEAC, mmol/L)
P-Values: Group=0.966, BCS=0.530, Group:BCS=0.852
2.0
1.5
1.0
0.5
H
M
Ferric Reducing Antioxidant Power Assay (FRAP, mmol/L)
P-Values: Group=0.866, BCS=0.377, Group:BCS=0.930
4
3
2
1
H
M
Cupric Reducing Antioxidant Capacity in saliva (CUPRAC, mmol/
P-Values: Group=0.904, BCS=0.505, Group:BCS=0.950
1.5
1.0
0.5
0.0
H
M
Uric Acid in saliva (mg/dL)
P-Values: Group=0.110, BCS=0.002**, Group:BCS=0.152
0.45
0.40
0.35
0.30
0.25
H
M
Advanced Oxidation Protein Products in saliva (μmol/L)
P-Values: Group=0.191, BCS=0.374, Group:BCS=0.807
2500
2000
1500
1000
500
H
M

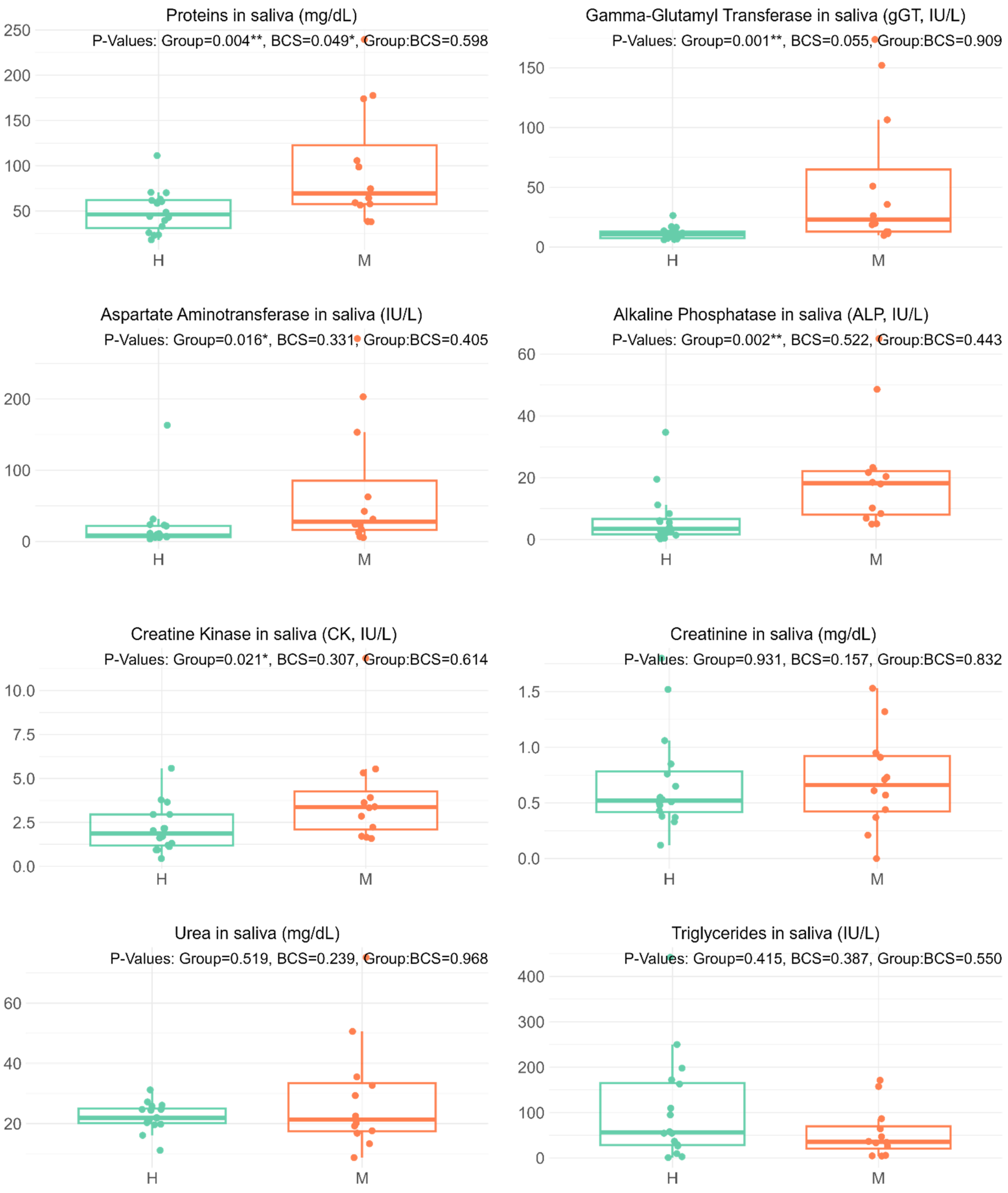
Proteins in saliva (mg/dL)
P-Values: Group=0.004**, BCS=0.049*, Group:BCS=0.598
H
M
Gamma-Glutamyl Transferase in saliva (gGT, IU/L)
P-Values: Group=0.001**, BCS=0.055, Group:BCS=0.909
H
M
Aspartate Aminotransferase in saliva (IU/L)
P-Values: Group=0.016*, BCS=0.331, Group:BCS=0.405
H
M
Alkaline Phosphatase in saliva (ALP, IU/L)
P-Values: Group=0.002**, BCS=0.522, Group:BCS=0.443
H
M
Creatine Kinase in saliva (CK, IU/L)
P-Values: Group=0.021*, BCS=0.307, Group:BCS=0.614
H
M
Creatinine in saliva (mg/dL)
P-Values: Group=0.931, BCS=0.157, Group:BCS=0.832
H
M
Urea in saliva (mg/dL)
P-Values: Group=0.519, BCS=0.239, Group:BCS=0.968
H
M
Triglycerides in saliva (IU/L)
P-Values: Group=0.415, BCS=0.387, Group:BCS=0.550
H
M

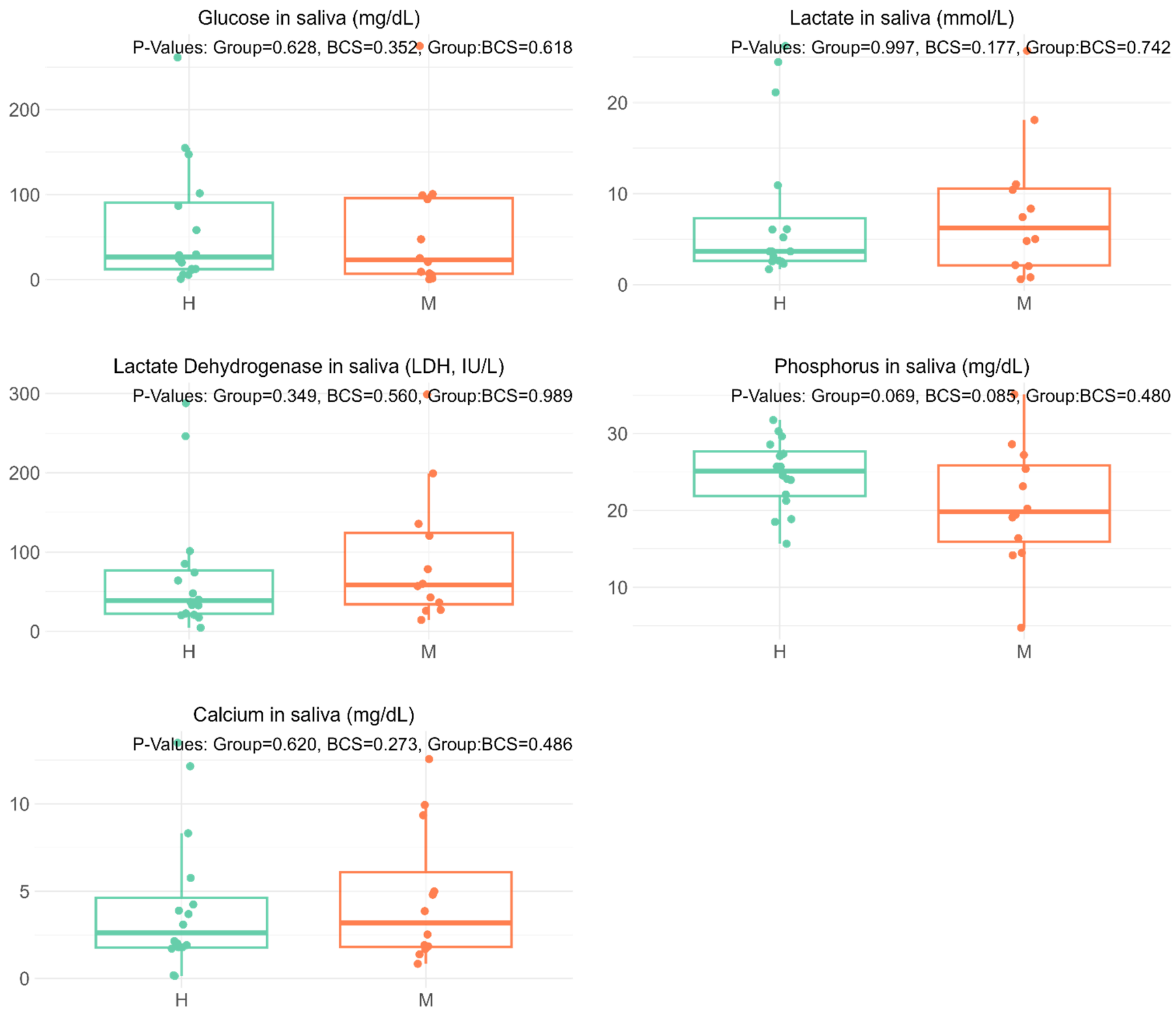


Figure S3. Results in saliva biomarkers of stress (cortisol, alpha-amylase, total esterase (TEA), lipase), immunity and inflammation (adenosine deaminase (ADA), haptoglobin (Hp)), oxidative status (Trolox equivalent antioxidant capacity (TEAC), ferric reducing ability of saliva (FRAS), cupric reducing antioxidant capacity (CUPRAC), uric acid, advanced oxidation protein products (AOPP)), enzymes (aspartate aminotransferase (AST), alkaline phosphatase (ALP), γ-glutamyl transferase (gGT), lactate dehydrogenase (LDH), creatine kinase (CK)), and proteins and minerals (creatinine, urea, triglycerides, glucose, lactate, proteins, phosphorus, calcium). Healthy controls are represented in green, while cows with metritis are represented in orange. The plots show the median and 25-75$^{th}$ percentile. Asterisk indicates the statistically significant differences between results (two-way analysis of variance (ANOVA), where "Group", "BSC" and the interaction “Group:BCS” are the factors considered in the analysis) (*: P < 0.05; **: P < 0.01).

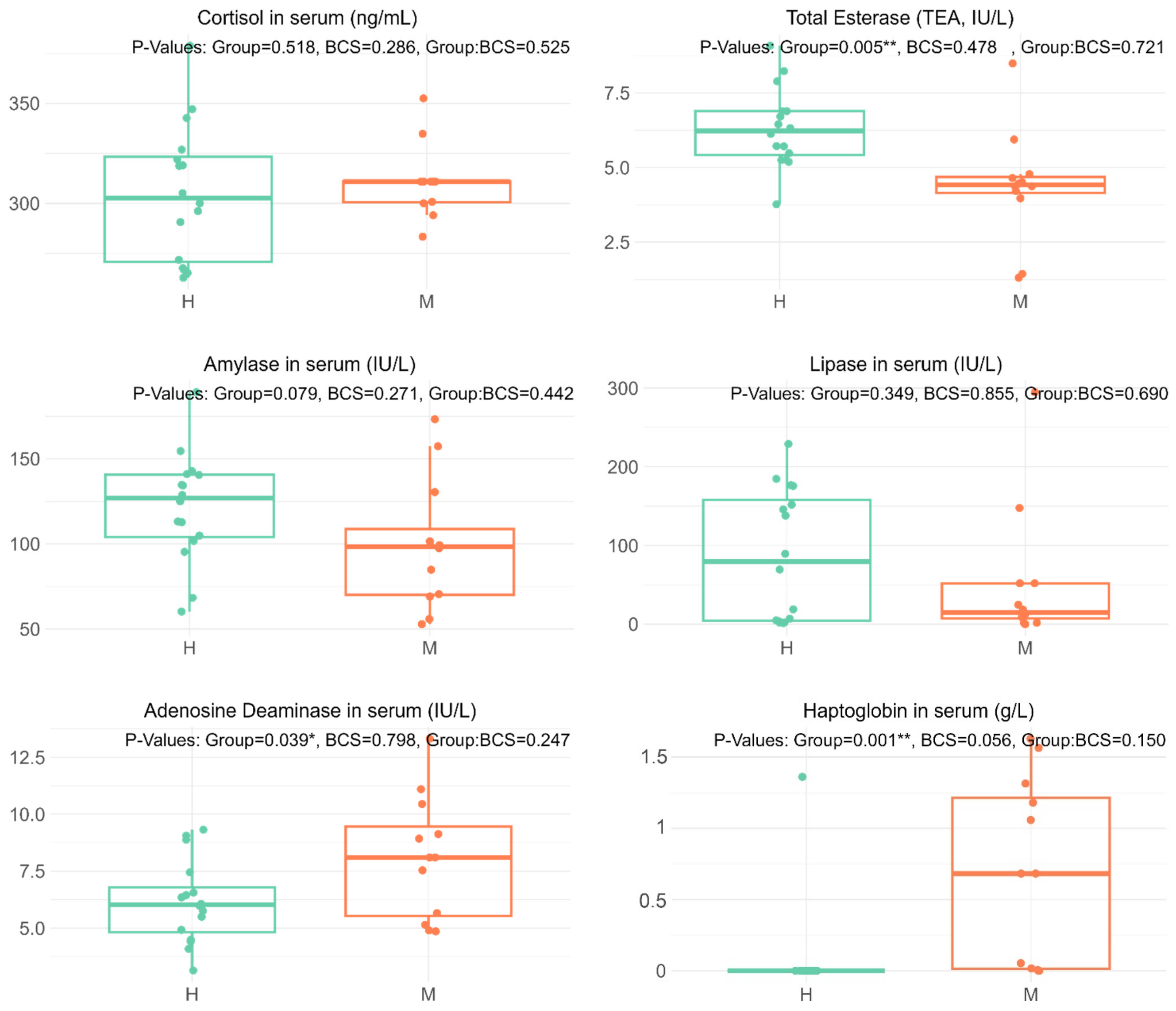
Cortisol in serum (ng/mL)
P-Values: Group=0.518, BCS=0.286, Group:BCS=0.525
350
300
H
M
Total Esterase (TEA, IU/L)
P-Values: Group=0.005**, BCS=0.478 , Group:BCS=0.721
7.5
5.0
2.5
H
M
Amylase in serum (IU/L)
P-Values: Group=0.079, BCS=0.271, Group:BCS=0.442
150
100
50
H
M
Lipase in serum (IU/L)
P-Values: Group=0.349, BCS=0.855, Group:BCS=0.690
300
200
100
0
H
M
Adenosine Deaminase in serum (IU/L)
P-Values: Group=0.039*, BCS=0.798, Group:BCS=0.247
12.5
10.0
7.5
5.0
H
M
Haptoglobin in serum (g/L)
P-Values: Group=0.001**, BCS=0.056, Group:BCS=0.150
1.5
1
0.5
0
H
M

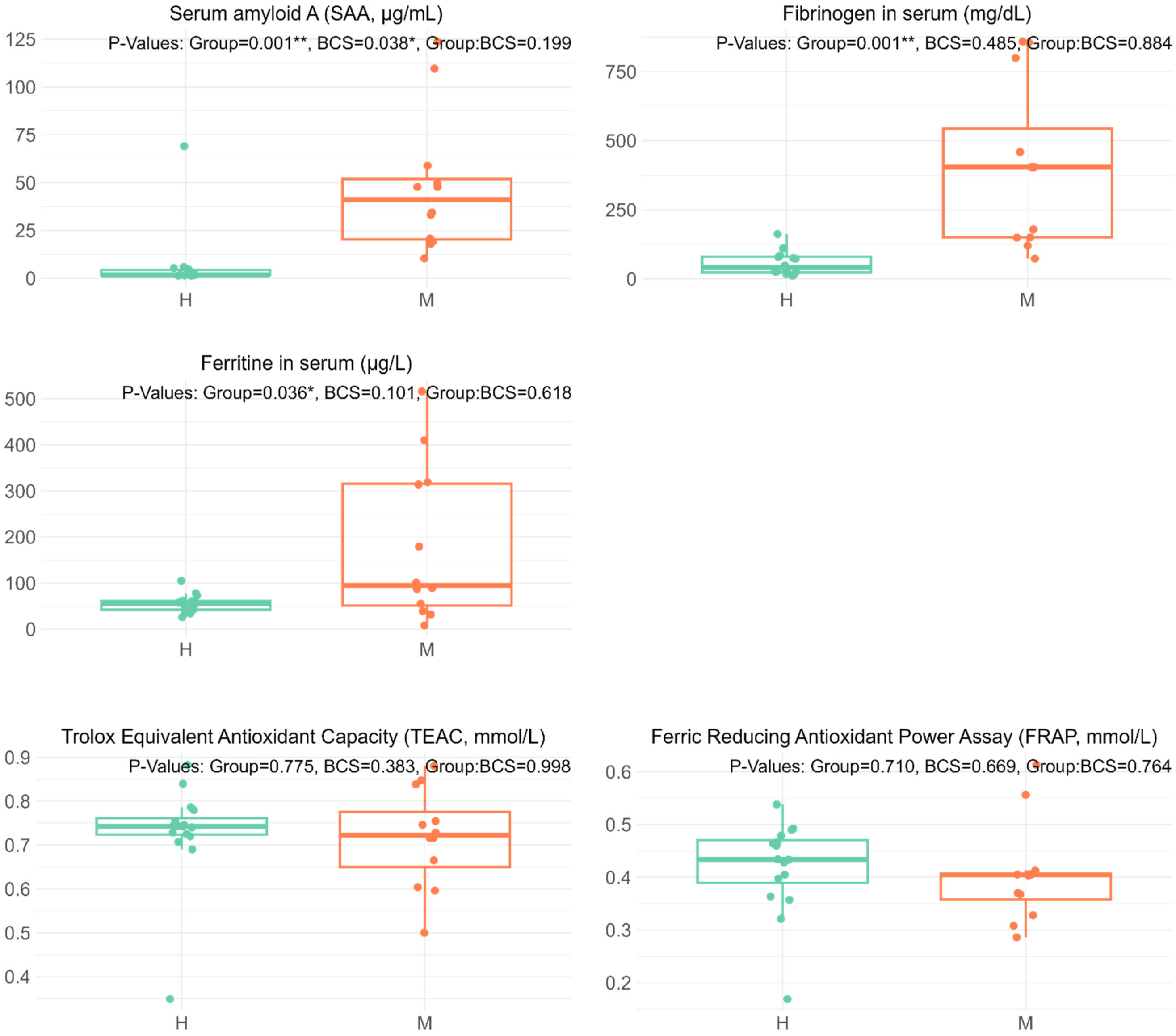

Serum amyloid A (SAA, µg/mL)
P-Values: Group=0.001**, BCS=0.038*, Group:BCS=0.199
125
100
75
50
25
0
H
M
Fibrinogen in serum (mg/dL)
P-Values: Group=0.001**, BCS=0.485, Group:BCS=0.884
750
500
250
0
H
M
Ferritine in serum (µg/L)
P-Values: Group=0.036*, BCS=0.101, Group:BCS=0.618
500
400
300
200
100
0
H
M
Trolox Equivalent Antioxidant Capacity (TEAC, mmol/L)
P-Values: Group=0.775, BCS=0.383, Group:BCS=0.998
0.9
0.8
0.7
0.6
0.5
0.4
H
M
Ferric Reducing Antioxidant Power Assay (FRAP, mmol/L)
P-Values: Group=0.710, BCS=0.669, Group:BCS=0.764
0.6
0.5
0.4
0.3
0.2
H
M

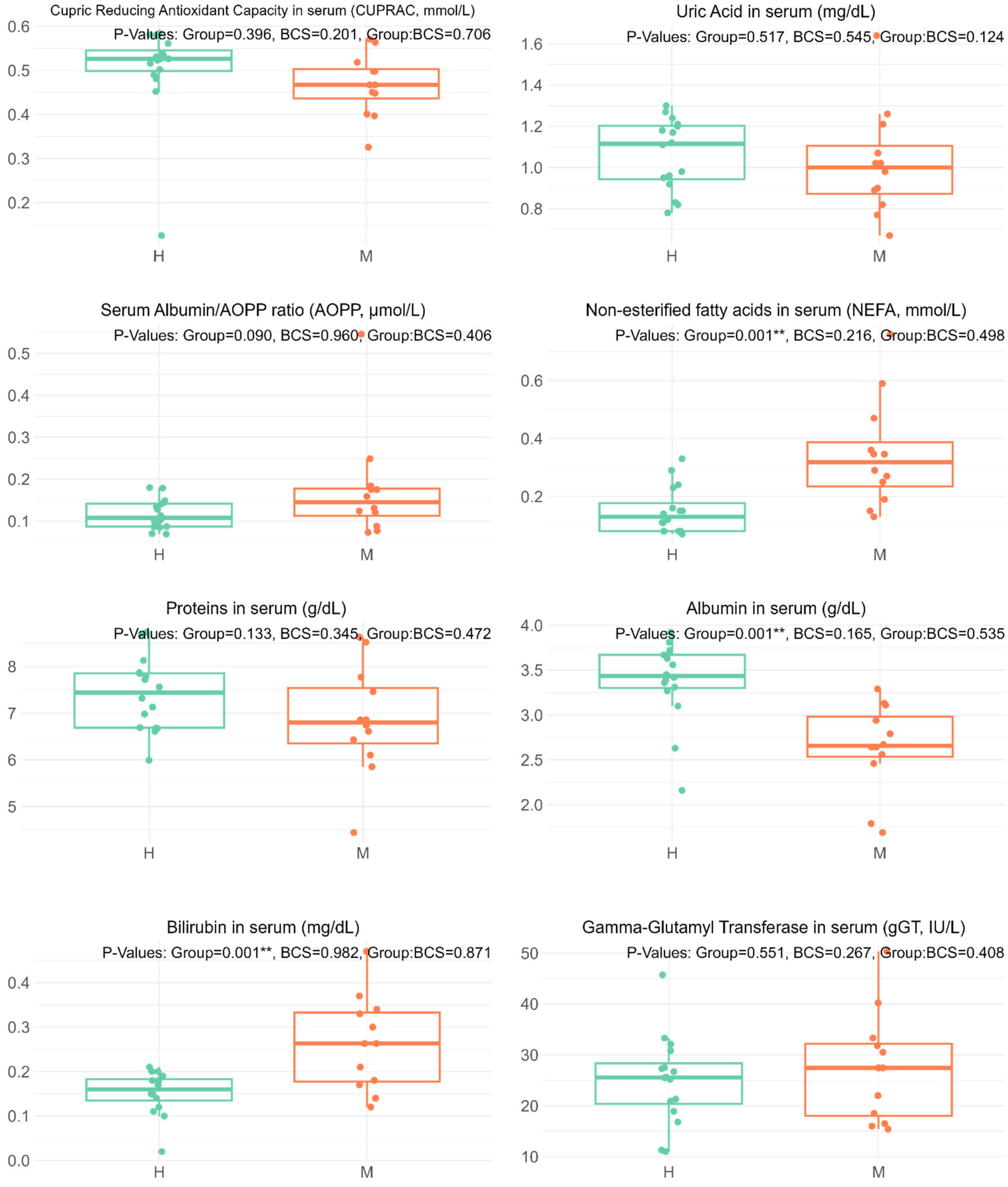
Cupric Reducing Antioxidant Capacity in serum (CUPRAC, mmol/L)
P-Values: Group=0.396, BCS=0.201, Group:BCS=0.706
0.6
0.5
0.4
0.3
0.2
H
M
Uric Acid in serum (mg/dL)
P-Values: Group=0.517, BCS=0.545, Group:BCS=0.124
1.6
1.4
1.2
1.0
0.8
H
M
Serum Albumin/AOPP ratio (AOPP, µmol/L)
P-Values: Group=0.090, BCS=0.960, Group:BCS=0.406
0.5
0.4
0.3
0.2
0.1
H
M
Non-esterified fatty acids in serum (NEFA, mmol/L)
P-Values: Group=0.001**, BCS=0.216, Group:BCS=0.498
0.6
0.4
0.2
H
M
Proteins in serum (g/dL)
P-Values: Group=0.133, BCS=0.345, Group:BCS=0.472
8
7
6
5
H
M
Albumin in serum (g/dL)
P-Values: Group=0.001**, BCS=0.165, Group:BCS=0.535
4.0
3.5
3.0
2.5
2.0
H
M
Bilirubin in serum (mg/dL)
P-Values: Group=0.001**, BCS=0.982, Group:BCS=0.871
0.4
0.3
0.2
0.1
0.0
H
M
Gamma-Glutamyl Transferase in serum (gGT, IU/L)
P-Values: Group=0.551, BCS=0.267, Group:BCS=0.408
50
40
30
20
10
H
M

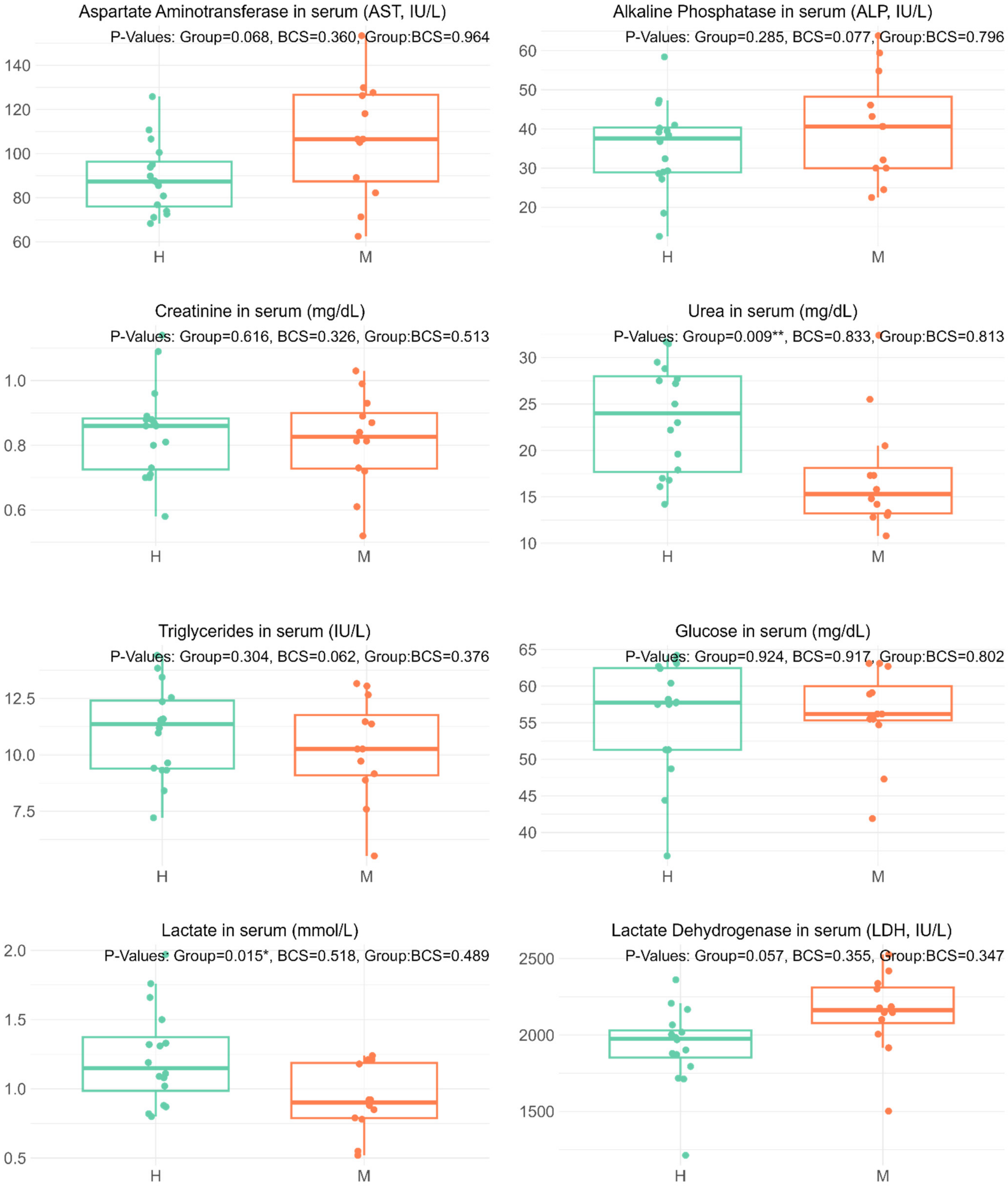
Aspartate Aminotransferase in serum (AST, IU/L)
P-Values: Group=0.068, BCS=0.360, Group:BCS=0.964
140
120
100
80
60
H
M
Alkaline Phosphatase in serum (ALP, IU/L)
P-Values: Group=0.285, BCS=0.077, Group:BCS=0.796
60
50
40
30
20
H
M
Creatinine in serum (mg/dL)
P-Values: Group=0.616, BCS=0.326, Group:BCS=0.513
1.0
0.8
0.6
H
M
Urea in serum (mg/dL)
P-Values: Group=0.009**, BCS=0.833, Group:BCS=0.813
30
25
20
15
10
H
M
Triglycerides in serum (IU/L)
P-Values: Group=0.304, BCS=0.062, Group:BCS=0.376
12.5
10.0
7.5
H
M
Glucose in serum (mg/dL)
P-Values: Group=0.924, BCS=0.917, Group:BCS=0.802
65
60
55
50
45
40
H
M
Lactate in serum (mmol/L)
P-Values: Group=0.015*, BCS=0.518, Group:BCS=0.489
2.0
1.5
1.0
0.5
H
M
Lactate Dehydrogenase in serum (LDH, IU/L)
P-Values: Group=0.057, BCS=0.355, Group:BCS=0.347
2500
2000
1500
H
M

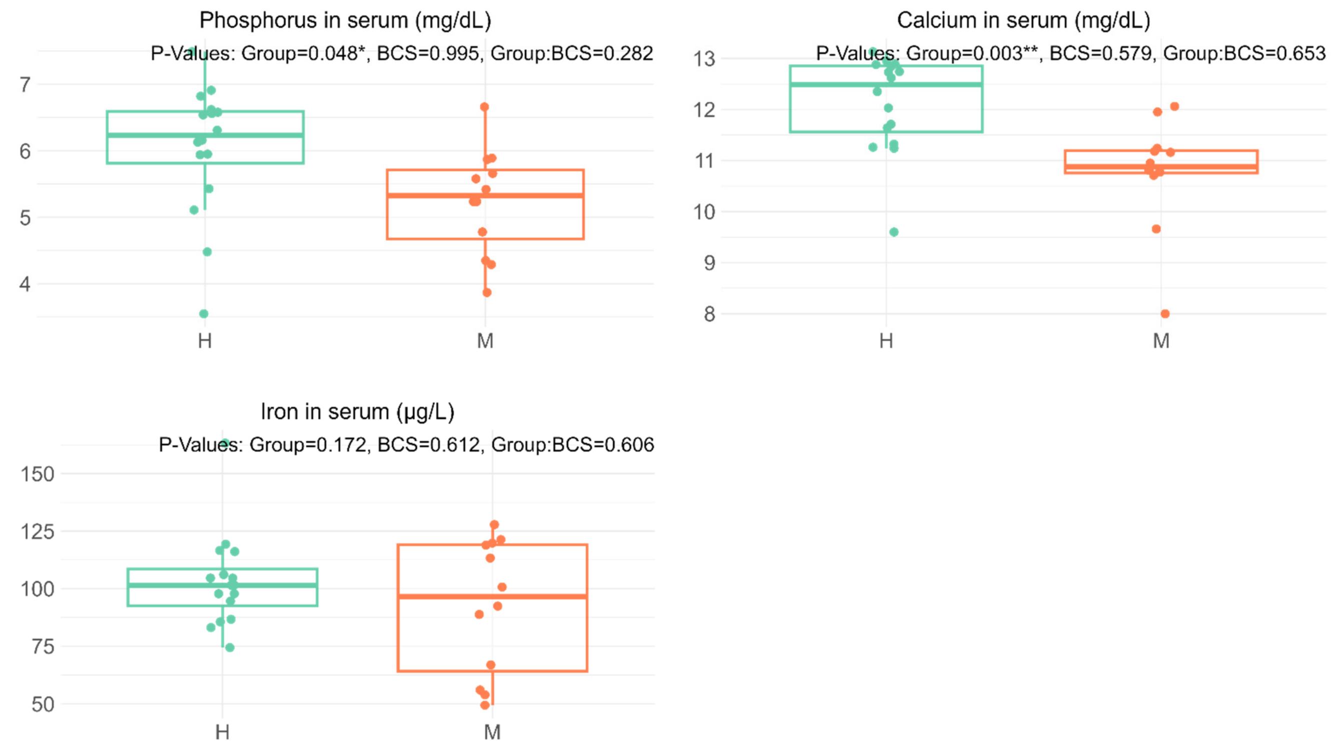


Figure S4. Results in serum biochemistry biomarkers of stress (cortisol, total esterase (TEA), amylase, lipase), immunity and inflammation (adenosine deaminase (ADA), haptoglobin (Hp), Serum Amyloid A (SAA), Fibrinogen, Ferritin), oxidative status (Trolox equivalent antioxidant capacity (TEAC), ferric reducing antioxidant power assay (FRAP), cupric reducing antioxidant capacity (CUPRAC), uric acid, advanced oxidation protein products/albumin ratio (AOPP/Albumin)), general metabolisms and enzymes (NEFA, albumin, bilirubin, aspartate aminotransferase (AST), alkaline phosphatase (ALP), γ-glutamyl transferase (gGT), lactate dehydrogenase (LDH), creatine kinase (CK)), and proteins and minerals (creatinine, urea, triglycerides, glucose, lactate, proteins, phosphorus, calcium and iron). Health controls are represented in green, while cows with metritis are represented in orange. Asterisk indicates the statistically significant differences between results (two-way analysis of variance (ANOVA), where "Group", "BSC" and the interaction “Group:BCS” are the factors considered in the analysis) (*: $P < 0.05$; **: $P < 0.01$).
